# SARS-CoV-2 biological clones are genetically heterogeneous and include clade-discordant residues

**DOI:** 10.1128/jvi.02250-24

**Published:** 2025-04-24

**Authors:** Ana Isabel de Ávila, María Eugenia Soria, Brenda Martínez-González, Pilar Somovilla, Pablo Mínguez, Llanos Salar-Vidal, Mario Esteban-Muñoz, Marta Martín-García, Sonia Zuñiga, Isabel Sola, Luis Enjuanes, Ignacio Gadea, Celia Perales, Esteban Domingo

**Affiliations:** 1 Centro de Biología Molecular “Severo Ochoa” (CSIC-UAM), Campus de Cantoblanco 16722, Madrid, Spain; 2 Department of Clinical Microbiology, Instituto de Investigación Sanitaria-Fundación Jiménez Díaz University Hospital, Universidad Autónoma de Madrid (IIS-FJD, UAM) 16722, Madrid, Spain; 3 Department of Molecular and Cell Biology, Centro Nacional de Biotecnología (CNB-CSIC), Consejo Superior de Investigaciones Científicas (CSIC), Campus de Cantoblanco, Madrid, Spain; 4 Departamento de Biología Molecular, Universidad Autonoma de Madrid, Campus de Cantoblanco 16722 , Madrid, Spain; 5 Department of Genetics and Genomics, Instituto de Investigación Sanitaria-Fundación Jiménez Díaz University Hospital, Universidad Autónoma de Madrid (IIS-FJD, UAM) 16722, Madrid, Spain; 6 Center for Biomedical Network Research on Rare Diseases (CIBERER), Instituto de Salud Carlos III 38176, Madrid, Spain; 7 Bioinformatics Unit, Instituto de Investigación Sanitaria-Fundación Jiménez Díaz University Hospital, Universidad Autónoma de Madrid (IIS-FJD, UAM), Madrid, Spain; 8 Center for Biomedical Network Research on Infectious Diseases (CIBERINFEC) 637284 , Madrid, Spain; The Ohio State University, Columbus, Ohio, USA

**Keywords:** RNA virus, quasispecies, mutant spectrum, viral clade, defective viral genome, population complexity, ultra-deep sequencing, diversity indices, point mutation, deletion

## Abstract

Defective genomes are part of SARS-CoV-2 quasispecies. High-resolution, ultra-deep sequencing of bulk RNA from viral populations does not distinguish RNA mutations, insertions, and deletions in viable genomes from those in defective genomes. To quantify SARS-CoV-2 infectious variant progeny, virus from four individual plaques (biological clones) of a preparation of isolate USA-WA1/2020, formed on Vero E6 cell monolayers, was subjected to further biological cloning to yield 9 second-generation and 15 third-generation sub-clones. Consensus genomic sequences of the biological clones and sub-clones included an average of 2.8 variations per viable genome, relative to the consensus sequence of the parental USA-WA1/2020 virus. This value is 6.5-fold lower than the estimates for biological clones of other RNA viruses such as bacteriophage Qβ, foot-and-mouth disease virus, or hepatitis C virus in cell culture. The mutant spectrum complexity of the nsp12 (polymerase)- and spike (S)-coding region was unique in the progeny of each of 10 third-generation sub-clones; they shared 2.4% of the total of 164 different mutations and deletions scored in the 3,719 genomic residues that were screened. The presence of minority out-of-frame deletions revealed the ease of defective genome production from an individual infectious genome. Several low-frequency point mutations and deletions were clade-discordant in that they were not typical of USA-WA1/2020 but served to define the consensus sequences of future SARS-CoV-2 clades. Implications for SARS-CoV-2 adaptability and COVID-19 control of the viable genome heterogeneity and the generation of complex mutant spectra from individual genomes are discussed.

**IMPORTANCE:**

Sequencing of biological clones is a means to identify mutations, insertions, and deletions located in viable genomes. This distinction is particularly important for viral populations, such as those of SARS-CoV-2, that contain large proportions of defective genomes. By sequencing biological clones and sub-clones, we quantified the heterogeneity of the viable complement of USA-WA1/2020 to be lower than exhibited by other RNA viruses. This difference may be due to a reduced mutation rate or to limited tolerance of the large coronavirus genome to incorporate mutations and deletions and remain functional or a combination of both influences. The presence of clade-discordant residues in the progeny of individual biological sub-clones suggests limitations in the occupation of sequence space by SARS-CoV-2. However, the complex and unique mutant spectra that are rapidly generated from individual genomes suggest an aptness to confront selective constraints.

## INTRODUCTION

Quasispecies dynamics within infected individuals underlie the short-term adaptive capacity of RNA viruses and condition the disease control strategies intended to minimize the selection of vaccine- or antiviral agent-escape mutants (recently reviewed in references 1–5). Initial suggestions were that SARS-CoV-2 displayed limited intra-host evolution (6). However, for this emerging human viral pathogen, there is extensive evidence of intra-host genetic and functional heterogeneity, quasispecies compartmentalization, and modifications of the mutant spectrum composition in sequential viral samples from immunocompetent and immunocompromised COVID-19 patients (3, 7–26).

Several factors might have influenced the non-unanimous view on SARS-CoV-2 intra-host diversity. (i) Different capacities to detect low-frequency mutations and deletions present in the mutant spectra, depending on the ultra-deep sequencing protocols. (ii) The assumption that the virus-coded 3′−5′ exonuclease (Exo N), which is active *in vitro* (27–29), would limit mutational input during viral replication, as it was previously documented with the coronavirus murine hepatitis virus (30). Yet, no calculation of the decrease in SARS-CoV-2 error rate that can be attributed to Exo N has been reported. (iii) Estimates of SARS-CoV-2 mutation rate (31, 32) are on the low side of the range of values reported for other RNA viruses (33–35), which fits the notion that Exo N may correct part of the erroneous (non-complementary to the template) nucleotides introduced in the nascent viral RNA product.

An additional uncertainty arises from the impossibility of assigning mutations and deletions (or other less frequent RNA modifications such as insertions, duplications, or inversions) to either viable genomes or defective viral genomes (DVGs) when subjecting unfractionated viral RNA populations to high-resolution ultra-deep sequencing protocols. This recognized problem (36) is fully manifested when amplicons of limited length are required to attain a mutation and deletion frequency cut-off of 0.1% (19, 20); mutations in a haplotype can be assigned to a DVG only when they are accompanied by out-of-frame (frameshift) deletions that lead to a protein synthesis termination codon or a lethal point mutation (i.e., one that abolishes the catalytic site of an essential enzyme).

The mapping of mutations and deletions in viable genomes is particularly relevant for SARS-CoV-2 since populations of this virus accumulate high frequencies of DVGs (37–40). DVGs have been involved in induction of the innate immune response, in modulation of replication of the corresponding standard (infectious or helper) genomes, in regulation of translation shut-off, in building reservoirs of variant sequences, in establishment of persistent infections, and in facilitating the transition toward extinction in lethal mutagenesis of viruses (39, 41–51). DVGs can also be components of pathological infectious units (52). *Trans*-complemented, RNA replication-competent DVGs do not need to express functional versions of some of the proteins that participate in the infectious cycle. Such DVGs may be more tolerant to mutations and deletions than their viable counterparts, thus inflating overall complexity measures when bulk viral RNA is analyzed.

In the present study, we have sequenced the SARS-CoV-2 RNA genome of biological clones and sub-clones retrieved from isolate USA-WA1/2020 (53), with the objective of quantifying the genetic lesions in the infectious virus subset of the population. We base the approach on the evidence that dilute virus passage and plaque isolation are effective means to purge viral populations of DVGs (48, 54, 55). Accordingly, a SARS-CoV-2 USA-WA1/2020 preparation was subjected to sequential plaque isolations on Vero E6 cell monolayers, with each isolation being preceded by a mild detergent treatment to favor particle disaggregation. The term clone denotes four biological clones (plaque isolates) retrieved from preparation of USA-WA1/2020. The term sub-clone refers to biological clones obtained from the four initial clones; they are divided into second- and third-generation sub-clones that result from two or three successive plaque isolations, respectively. The genomic sequence of the virus was directly punched from each of a total of 28 individual plaques (without further infectious rounds) revealed multiple point mutations and deletions, either unique or shared by several clones or sub-clones. The genetic heterogeneity among viable SARS-CoV-2 genomes is lower than previously estimated for biological clones of bacteriophage Qβ (56), foot-and-mouth disease virus (FMDV) (57, 58), and hepatitis C virus (59). Ultra-deep sequencing of the nsp12 (polymerase)- and spike (S)-coding regions of third-generation sub-clonal populations revealed mutant clouds of lower complexity than those from patients’ nasopharyngeal samples. Despite their clonality, each of these mutant clouds displayed a unique and broad mutant spectrum, which includes frameshift deletions and clade-discordant residues (those that serve as signatures of SARS-CoV-2 clades other than the clade of USA-WA172020 isolate). The implications of these observations for SARS-CoV-2 evolution and COVID-19 management are discussed.

## RESULTS

### Preparation of clonal and sub-clonal populations of SARS-CoV-2 USA-WA1/2020

**Fig 1:**
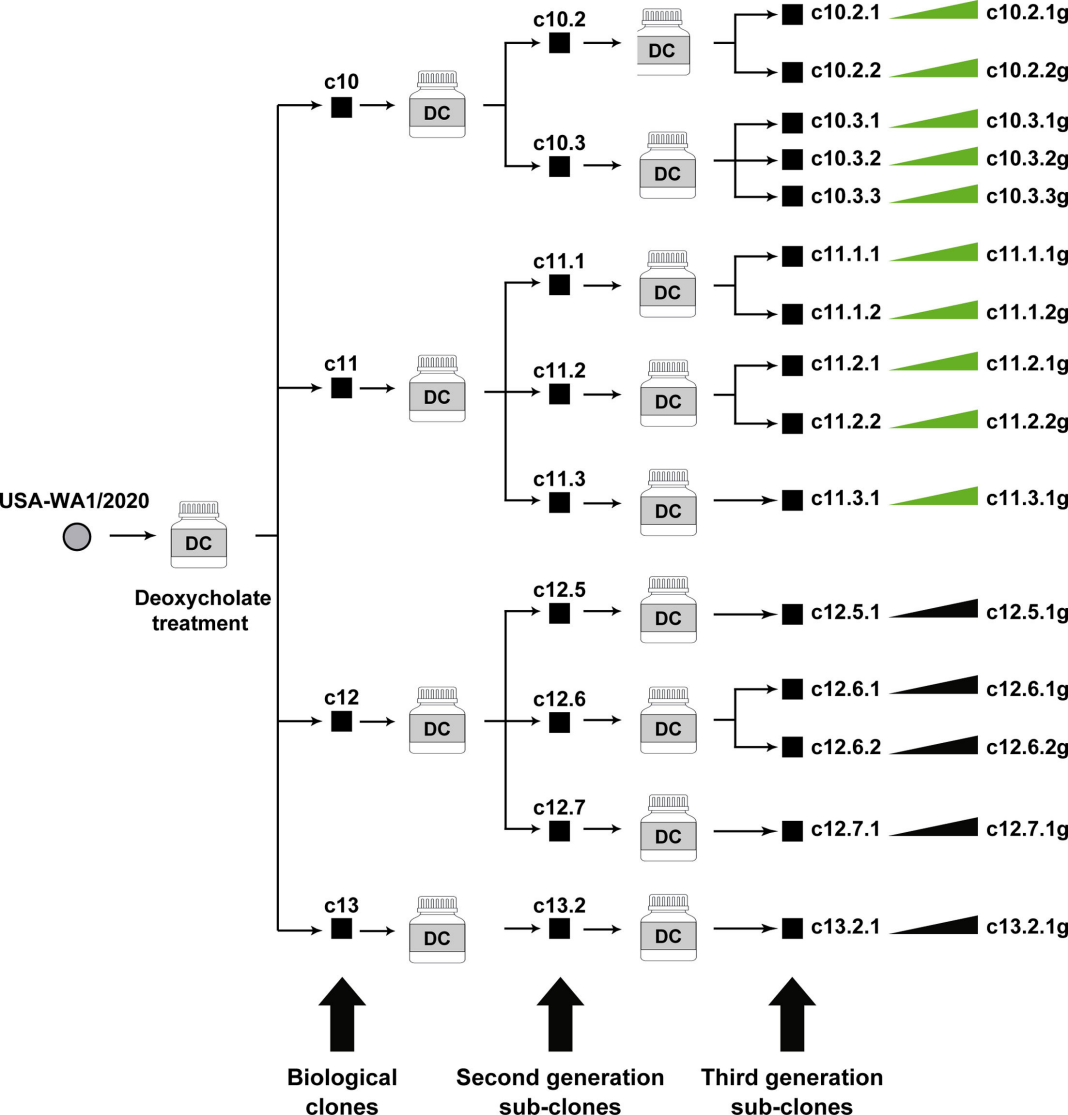
Schematic representation of the isolation of biological clones and sub-clones from SARS-CoV-2 USA-WA1/2020. The initial virus (circle on the left) was prepared by infection of Vero E6 cells of the virus received from BEI Resources. It was treated with deoxycholate (DC), diluted, and plated on Vero E6 cell monolayers to obtain biological clones c10, c11, c12, and c13 (filled squares). The process was repeated to obtain 9 second-generation and 15 third-generation sub-clones (indicated with horizontal arrows and filled squares and with arrows at the bottom). Virus from an aliquot of each of the third-generation sub-clones was grown in Vero E6 cells (represented by the elongated triangle and “g” following the sub-clone name; last row on the right of the scheme). RNA from the third-generation derivatives of clones c10 and c11 was analyzed by ultra-deep sequencing (green elongated triangles). Procedures are detailed in Materials and Methods.

The starting virus was SARS-CoV-2 USA-WA1/2020, prepared by infecting Vero E6 cells with the virus received from BEI Resources (www.beiresources.org); the origin and passage history of this isolate─that justifies the use of Vero E6 cells for this experiment─is described in reference 53 and summarized in Materials and Methods. Following preliminary experiments of resistance of USA-WA1/2020 infectivity to mild detergent treatment (Table S1 in https://saco.csic.es/s/kYsz6A4sbzssZRp), the virus was incubated with 0.01% deoxycholate in cell culture medium for 10 min at room temperature to disaggregate viral particles (57, 60); then, the mixture was diluted in culture medium and plated on Vero E6 cell monolayers. Material from four well-isolated plaques of average size was resuspended in a cell culture medium and subjected to identical deoxycholate treatment, dilution, and plating. The process was repeated with 9 second-generation sub-clones to yield 15 third-generation sub-clones (Fig. 1).

Some differences were noted in infectious titer, amount of viral RNA, and specific infectivity among the biological clones and sub-clones (data in Fig. S1 in https://saco.csic.es/s/kYsz6A4sbzssZRp). In all cases, sufficient viral RNA was obtained to proceed with the determination of the consensus nucleotide sequence of genomic RNA directly from the virus resuspended from a viral plaque, without the need for additional infection rounds.

### Mutations and deletions in the consensus genomic sequence of clonal and sub-clonal populations

**Fig 2:**
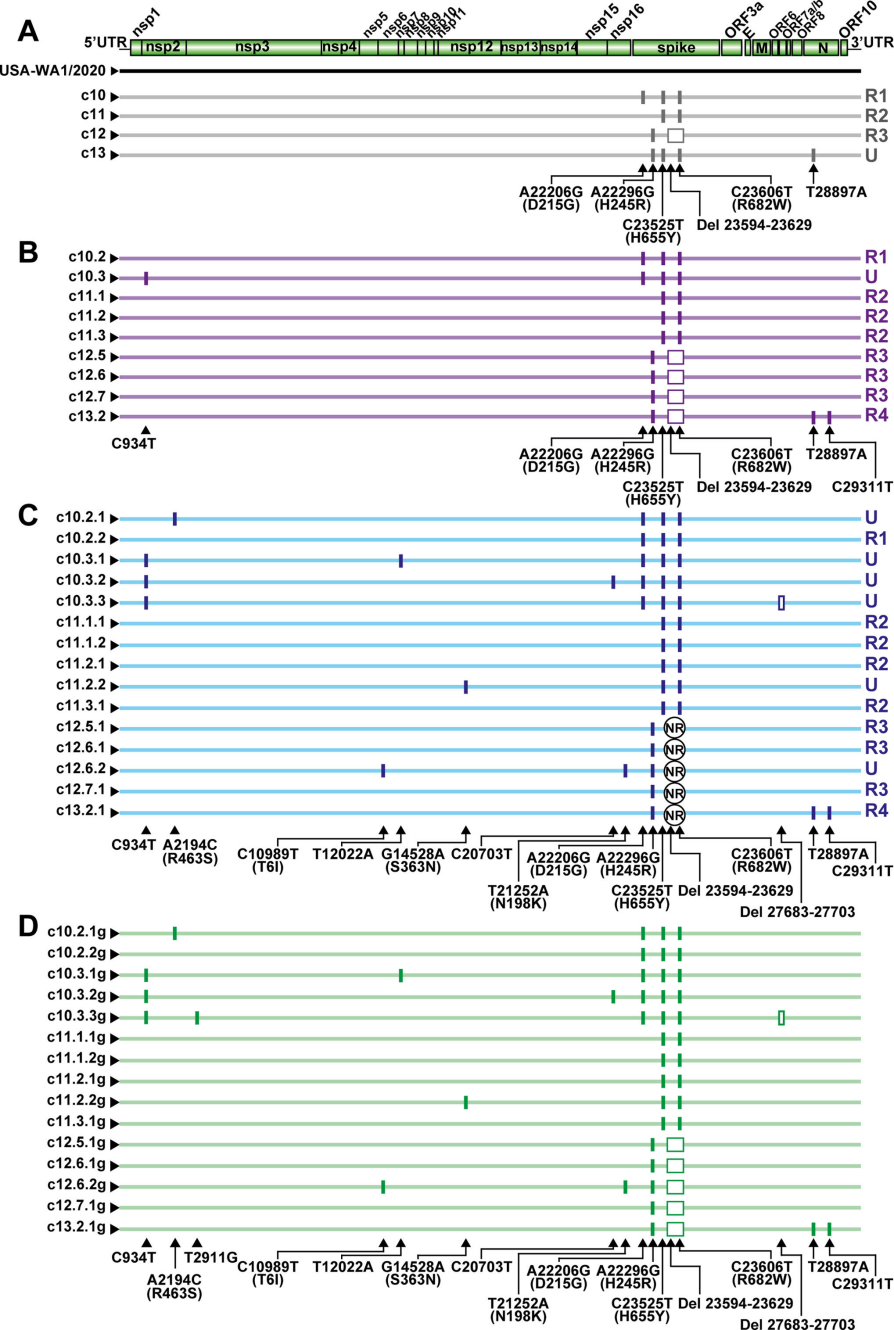
Alignment of consensus genome sequences of biological clones and sub-clones. (**A**) A scheme of the SARS-CoV-2 genome with indication of viral proteins and the 5′, 3′ untranslated regions (UTR) depicted at the top. Below, the genome of the USA-WA1/2020 (used as reference) and of the initial biological clones is indicated with horizontal lines. The virus clone is given at the left of each line. (**B and C**) Same as A but with second- and third-generation sub-clones. NR means non-resolved sequences; they involve 35 nucleotides located between residues 23480 and 23628. (**D**) Same as C but for the third generation sub-clones further grown in Vero E6 cells (name of sub-clone followed by g). Mutations are indicated with vertical lines and deletions by rectangles. The mutated residues (with the amino acid substitution for non-synonymous mutations indicated in parentheses) and the deletions (Del) are given at the bottom of each of the alignments in A, B, C, and D (with T representing U in the RNA); residue numbering is that of reference sequence Wuhan-Hu-1 (NC_045512.2). For A, B, and C genomes with a unique or repeated sequence, considering all clones and sub-clones, are distinguished at the right of each line, as follows; U, unique genomic sequence; R1, a sequence repeated in three genomes; R2, a sequence repeated in eight genomes; R3, a sequence repeated in seven genomes; R4, a sequence repeated in two genomes. Note that genomes in blocks C and D differ only in a synonymous mutation (T2911G). The procedure used for the determination of consensus sequences is explained in Materials and Methods.

To estimate the genetic heterogeneity among the viable subset of genomes in the USA-WA1/2020 preparation, the consensus genomic nucleotide sequence of four biological clones (c10, c11, c12, and c13 in Fig. 1) was determined using the COVIDSeq sequencing (Illumina). Alignment of the sequences with that of the parental USA-WA1/2020 preparation (taken as reference for the counting of mutations and deletions) revealed an average of 2.5 point mutations and 0.2 deletions per genome (Fig. 2A). To calculate the viable genome heterogeneity within each clone and in subsequent sub-clones, the consensus genomic nucleotide sequence of 9 second-generation and 15 third-generation sub-clones (Fig. 1) was determined. Sequence alignment taken as reference for the sequence of USA-WA1/2020 revealed an average of 2.1 point mutations and 0.4 deletions per genome for the second-generation sub-clones and 2.7 point mutations and 0.4 deletions per genome for the third-generation sub-clones. For sub-clones c12.5.1, c12.6.1, c12.6.2, c12.7.1, and c13.2.1, a stretch of 35 nucleotides was unresolved; in all cases, it was located between residues 23480 and 23628 (indicated as non-resolved [NR] in Fig. 2). For mutation and deletion counting, we have taken the conservative assumption that the sequence of the NR residues is identical to that of the corresponding parental sub-clones and progeny populations (compare Fig. 2B, C and D). In the second and third sub-clonal lineages, the following number of new genetic lesions were counted: four point mutations and one deletion for the c10 lineage; one point mutation for lineage c11; two point mutations for lineage c12; two point mutations (one of them a reversion); and a deletion for lineage c13. The only variation that crossed lineages was Del 23594–23629 (from lineage c12 to c13); this deletion spans the furin cleavage site in S, and it may be a frequent lesion in virus replicating in Vero E6 cells (see Discussion). The number of mutations and deletions in the consensus sequence, located between genomic residues 22206 and 23629 (S amino acids 215–689) of the biological clones and sub-clones analyzed, may qualify this stretch as a hot spot of variation, relative to the rest of the genome. In that genomic stretch, the average number of lesions was 1.4 × 10^−3^ lesions per nucleotide and clone. The corresponding number for the rest of the genome is 2.0 × 10^−5^ lesions per nucleotide and clone (*P* < 0.001; proportion test).

The step-wise accumulation of mutations and deletions in each lineage is depicted in Fig. S2 in https://saco.csic.es/s/kYsz6A4sbzssZRp. Of the total 28 genomes from clones and sub-clones analyzed, 8 had a unique sequence (indicated as U in Fig. 2), while the other sequences were repeated several times (indicated as R1, R2, R3, and R4 in Fig. 2). Upon further growth of clones c10.2.1 to c13.2.1 to yield c10.2.1g to c13.2.1g, respectively, only one synonymous mutation (U2911G) was added to the consensus sequence of c10.3.3g (Fig. 2C and D), yielding an average of 3.2 variations per genome. Therefore, the viable moiety of the USA-WA1/2020 preparation was genetically heterogeneous, and individual clones and sub-clones yielded viable genomes with additional variations in the course of plaque development on Vero E6 cells.

### Kinetics of virus progeny production and mutant spectrum complexity of sub-clonal populations

**Fig 3:**
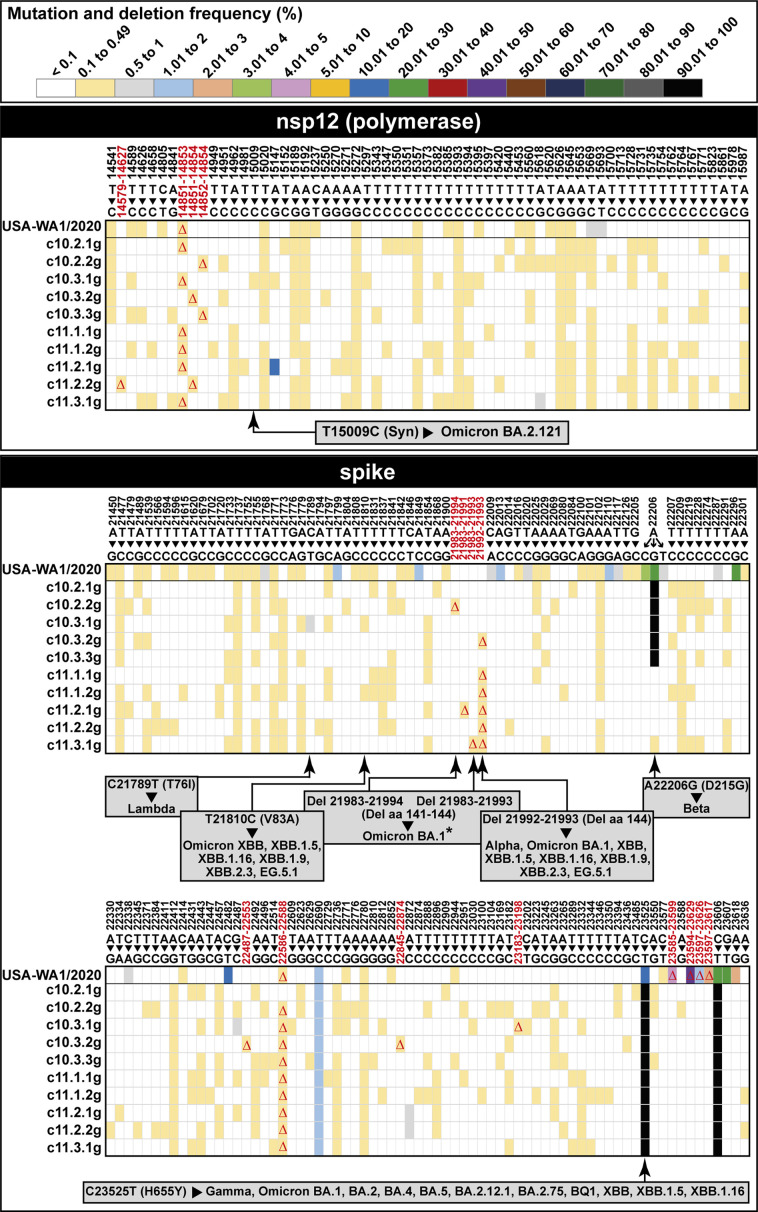
Heat map of point mutations and deletions identified in the mutant spectrum of USA-WA1/2020 and 10 biological sub-clones. Values of mutation frequency are color coded as shown in the top box. The genomic region analyzed is indicated in the filled rectangle at the top of each panel group. The nsp12 (polymerase) region analyzed spans residues 14534–16054, and the spike (S)-coding region analyzed spans residues 21448–23645. The name of the virus or biological sub-clone is given at the left of each line within a panel. Only positions where mutations or deletions have been found are included in the map. Deletions are indicated with triangles. Residues are numbered according to reference sequence Wuhan-Hu-1 (NC_045512.2). Clade-discordant mutations and deletions are indicated inside gray rectangles. In them, amino acid substitutions are given in parentheses following the corresponding point mutation. Del and Del aa indicate deletions of nucleotides and amino acids, respectively; the asterisk in Omicron BA.1 means that in that viral lineage, the amino acids deleted are 142–144. The clade-discordant residues are also indicated in Table S6 (https://saco.csic.es/s/kYsz6A4sbzssZRp), where all mutations and deletions found in the mutant spectra and in the consensus sequences are listed. Procedures for ultra-deep sequencing and controls for reliability of mutation and deletion detection are explained in Materials and Methods.

We previously documented a great complexity of the SARS-CoV-2 mutant spectrum of the nsp12 (polymerase)- and spike (S)-coding regions of nasopharyngeal isolates from COVID-19 patients of different disease severity (18, 19), as well as of USA-WA1/2020 populations passaged in Vero E6 cells, in the absence or presence of mutagenic nucleoside analogs (61, 62). These quantifications provide a basis for comparison with the complexity of the sub-clones isolated in the present study. To this aim, virus from 10 third-generation sub-clones derived from c10 and c11 (sub-clones c10.2.1 to c11.3.1 in Fig. 1) was used to infect Vero E6 cells to produce the amount of viral RNA required for high-resolution Illumina MiSeq ultra-deep sequencing (sub-clones c10.2.1g to c11.3.1g in Fig. 1). The kinetics of viral production were very similar for each of the sub-clones and for the parental USA-WA1/2020 population (*P* > 0.99 in all comparisons; two-way analysis of variance (ANOVA); Fig. S3 in https://saco.csic.es/s/kYsz6A4sbzssZRp). Prior to entering the ultra-deep sequencing protocol, the amplification of serial dilutions of the extracted RNA ensured that there was no limitation in the initial amount of template molecules that could bias mutant spectrum composition for comparative purposes (procedures, oligonucleotide primers, and controls for reliability of mutation and deletion detection are described in Materials and Methods). A heat map of mutations and deletions in the nsp12 (polymerase)- and spike (S)-coding region, determined with a 0.1% frequency cut-off, indicates that each mutant spectrum had a unique composition, with the great majority of mutations found in the 0.10%–0.49% frequency range (Fig. 3). Out of the 3,719 positions screened by the amplicons, 333 positions (8.9%) were mutated or deleted in the mutant spectrum of the sub-clones; 6 out of 149 mutations (4%) were shared by all sub-clones. Three out of the six mutations that were present in the 10%–50% frequency range in the USA-WA1/2020 preparation were dominant in the consensus sequence of biological clones and sub-clones; these mutations and corresponding amino acid substitutions were: A22206G (S substitution D215G), C23525T (S substitution H655Y), and C23606T (S substitution R682W). Six out of 16 deletions were out of frame, indicating the ease of production of defective genomes, following a limited number of replication rounds (Table S2 in https://saco.csic.es/s/kYsz6A4sbzssZRp).

**Fig 4:**
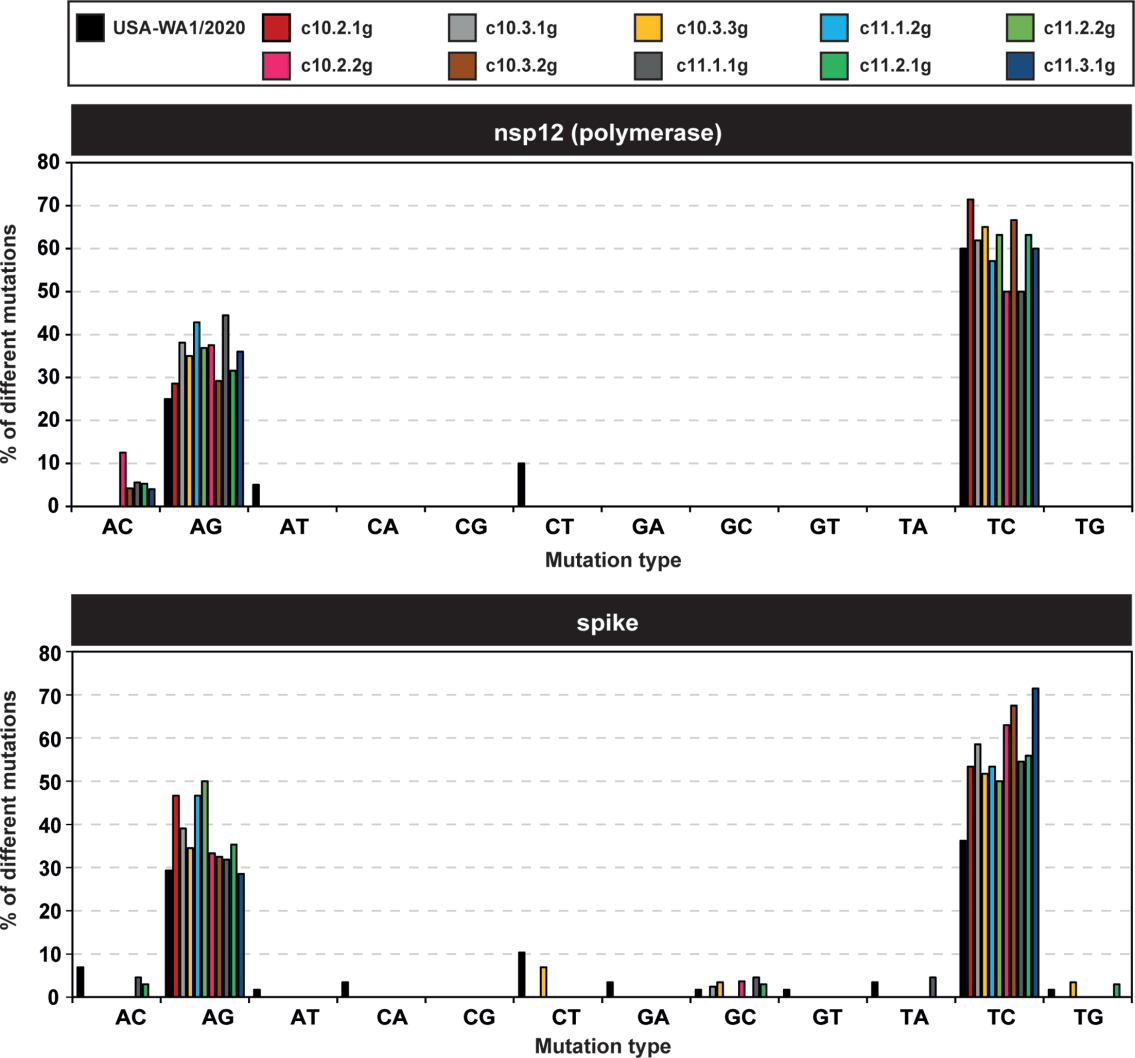
Distribution of mutation types in the mutant spectrum of nsp12 (polymerase) and S amplicons of USA-WA1/2020 and biological sub-clones. Viruses are identified by a color code depicted in the upper box. Mutations are counted relative to the consensus sequence of the corresponding amplicon. Mutation types are displayed in abscissa, and the percentage of each mutation type is given in ordinate. The location of the amplicons in the SARS-CoV-2 genome, and the ultra-deep sequencing procedure are detailed in Materials and Methods.

In contrast to the mutation type preferences in consensus sequences, the mutant spectra of the nsp12 (polymerase)- and S-coding region of biological sub-clones exhibited an overwhelming dominance of T → C and A → G transitions (Fig. 4). The origin of the different mutation type preferences in consensus sequences vs mutant spectra is not understood (see Discussion for possible contributing factors).

**Fig 5:**
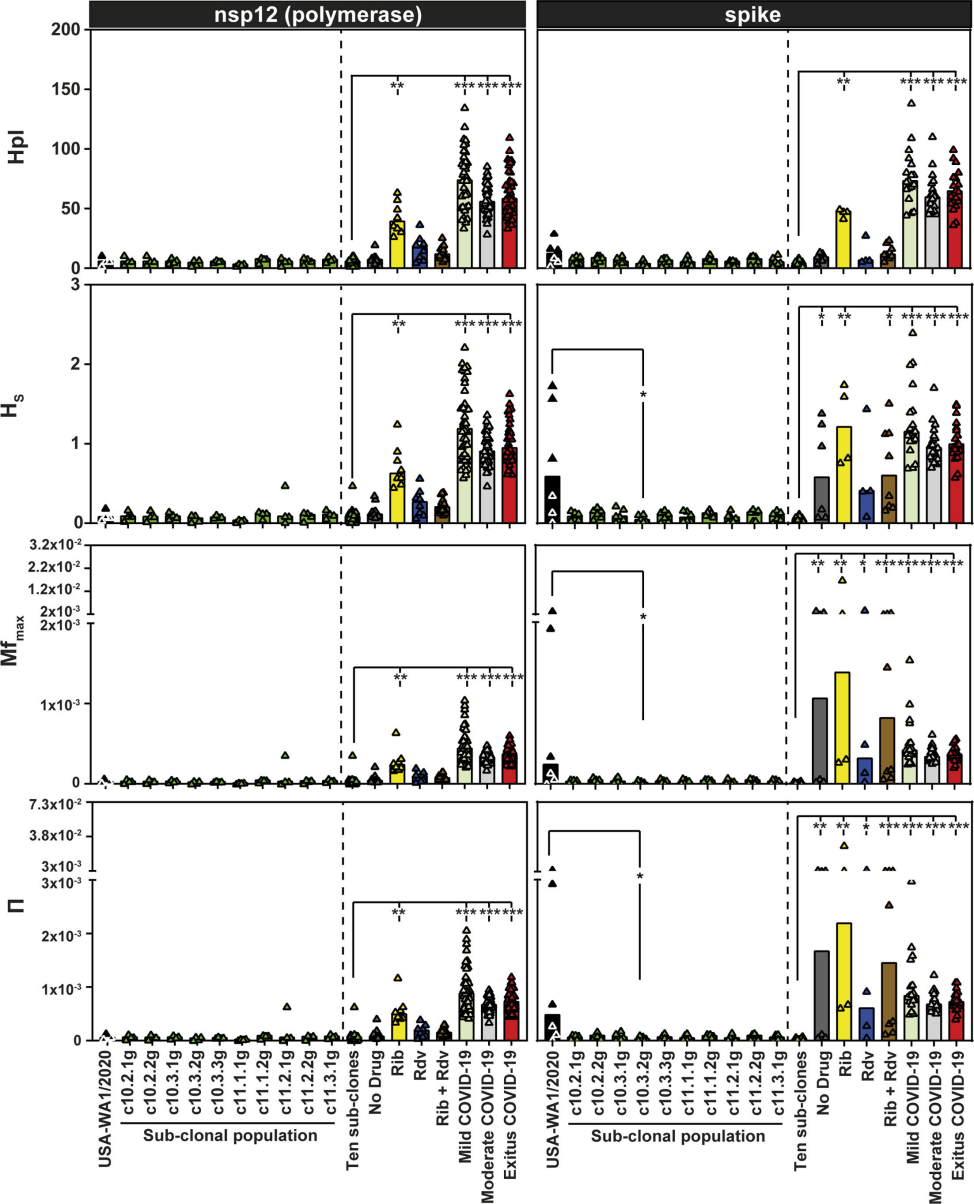
Diversity indices of the mutant spectrum of SARS-CoV-2 USA-WA1/2020, its biological sub-clones, laboratory populations, and nasopharyngeal isolates of the virus. The top filled boxes indicate the genomic region analyzed. The diversity index is given in ordinate (Hpl, number of haplotypes; Hs, Shannon entropy; Mf_max_, maximum mutation frequency; *ᴨ*, nucleotide diversity) (63). The name of the virus, sub-clone, or laboratory population is written in the abscissa: Ten sub-clones: aggregate of values of the 10 sub-clones. No drug: laboratory population passaged in Vero E6 cells in the absence of drug. Rib: laboratory population passaged in Vero E6 cells in the presence of ribavirin. Rdv: laboratory population passaged in Vero E6 cells in the presence of remdesivir. Rib + Rdv: laboratory population passaged in Vero E6 cells in the presence of combinations of ribavirin and remdesivir. Laboratory populations are described in references 61, 62. Mild, moderate, and exitus COVID-19 correspond to the virus from nasopharyngeal isolates of patients with different disease severity described in reference 19. Bars represent the median for the nsp12 (polymerase) and spike (S) amplicons; triangles are the values for each sample and amplicon. Statistical differences have been calculated using the Kruskal-Wallis test, followed by Dunn’s multiple comparison test (*, *P* < 0.05; **, *P* < 0.01; ***, *P* < 0.001). Additional diversity indices for the same samples are given in Fig. S4, and the numerical values for each sample and amplicon are compiled in Tables S4 and S5 in https://saco.csic.es/s/kYsz6A4sbzssZRp or in the references quoted therein. Amplicon residues and procedures are described in Materials and Methods.

Abundance and incidence diversity indices that are used to quantify quasispecies complexity (63) indicate that USA-WA1/2020 and the biological sub-clones exhibit a uniform and significantly lower complexity than the multiply passaged laboratory populations or nasopharyngeal isolates of SARS-CoV-2 (Fig. 5 and Fig. S4; Tables S3 and S4 in https://saco.csic.es/s/kYsz6A4sbzssZRp). This difference is likely due to the limited diversification of the biological sub-clones (it took about 27 infectious genome doublings to go from an initial infectious genome to the populations analyzed by ultra-deep sequencing [see Fig. S1 in https://saco.csic.es/s/kYsz6A4sbzssZRp]). A tendency (that in some cases reached statistical significance) was noted toward higher complexity of USA-WA1/2020 than its derived sub-clones in the S-coding region but not in the nsp12 (polymerase)-coding region (see Discussion for two interpretations of the difference in complexity between the two genomic regions). Thus, SARS-CoV-2 biological sub-clones are genetically complex but to a lesser degree than nasopharyngeal samples from COVID-19 patients, indicating that mutant spectrum complexity is a variable trait, influenced by the evolutionary history of the virus.

### Clade-discordant residues in USA-WA1/2020 and its sub-clonal derivatives

Previous work showed that mutant spectra of SARS-CoV-2 contained mutation and deletion clusters that were not typical of the clade of the isolate under study but that were sequence signatures that helped to define the clade of future dominant viruses (14, 21, 64). Since the origin of such clade-discordant sequences was not clear, it was interesting to investigate whether they were also present in the biological sub-clones under study. At the time of isolation of USA-WA1/2020, no other SARS-CoV-2 clades were circulating in the world (53). In all sub-clones, at least two clade-discordant mutations or deletions were identified that were typical of Alpha, Beta, Gamma, Lambda, or Omicron lineages (highlighted in Fig. 3 and in Table S5 in https://saco.csic.es/s/kYsz6A4sbzssZRp). The results indicate the rapid generation of clade-discordant residues upon limited replication of single SARS-CoV-2 genomes, suggesting that limitation in sequence space occupation facilitates their frequent occurrence.

The present study has quantified the frequency of genomic modifications in viable SARS-CoV-2 genomes and has indicated that complex mutant spectra develop even during limited rounds of virus multiplication. Furthermore, the analysis has reinforced the value of SARS-CoV-2 mutant spectra as repositories of sequences that become dominant in future viral clades.

## DISCUSSION

Quasispecies dynamics and the resulting population structure confer adaptability to viruses, with consequences for viral disease emergence and disease control (recently reviewed in references 1–5). Depending on the replication machinery and host components, some viruses, including SARS-CoV-2, produce DVGs at high frequency, many of which arise from intra-genome and inter-genome RNA recombination (65–69). In this scenario, the aim of the present study was to evaluate the number and types of genome variations produced in viable SARS-CoV-2 progeny in the controlled biological environment provided by Vero E6 cells. The increase from 1 PFU to the number of PFUs counted in the material resuspended from a plaque (clone or sub-clone; in the range of 10^4^ to 10^6^ PFU [Fig. S1 in https://saco.csic.es/s/kYsz6A4sbzssZRp]) is equivalent to 14–20 viable genome doublings. This is a modest number compared with the number that the virus likely undergoes *in vivo* from the initial infection until the collection of the diagnostic sample (70). The consensus genomic sequences of the clonal and sub-clonal populations include an average of 2.8 genomic modifications per viable genome, which corresponds to 0.09 genome modifications per 1,000 nucleotides. This heterogeneity is about 6.5-fold lower (range 2.5- to 14.4-fold) than the heterogeneity previously estimated for other RNA viruses: 1–2 mutations per genome, which equates to 0.23–0.47 mutations per 1,000 nucleotides for the bacteriophage Qβ genome (4,217 nucleotides) (56); 2–11 mutations per genome (0.24–1.30 mutations per 1,000 nucleotides) for the FMDV genome (8,400 nucleotides) (57, 58); 5.7 mutations per genome (0.58 mutations per 1,000 nucleotides) for the hepatitis C virus genome (9,600 nucleotides) (59). These calculations are summarized in Table S6 in https://saco.csic.es/s/kYsz6A4sbzssZRp. This difference may reflect a lower mutation rate or decreased tolerance of SARS-CoV-2 to mutations, as compared with other RNA viruses whose genomic RNA is three- to sevenfold shorter (2, 3, 71), or both. The presence of in-frame deletions in biological clones and sub-clones suggests that they are generated with high frequency during SARS-CoV-2 genome replication and tolerated among viable genomes.

It may be considered that in the course of plaque development, DVGs were generated and reached dominance so that the variations scored in the consensus genomic sequences belonged to DVGs rather than to infectious genomes. This is not compatible with genomic changes in biological clones being inherited by successive generation sub-clones (Fig. 2). The specific infectivity of the biological clones and sub-clones (Fig. S1 in https://saco.csic.es/s/kYsz6A4sbzssZRp) also argues against this possibility. Furthermore, serial plaque-to-plaque transfers lead to the accumulation of fitness-decreasing mutations (operation of Muller’s ratchet [58, 72]), suggesting a relaxed negative selection of genomic changes in the course of plaque development given the very small effective population size. Although a high multiplicity of infection may be reached locally during plaque formation (the area covered by a plaque includes about 800 cells that finally yield around 1 × 10^5^ PFUs [calculation described in Materials and Methods]), the limited intra-plaque competition among variants renders unlikely that DVGs could reach dominance by complementation. Still, another possibility is that despite DVGs not reaching dominance during plaque development, they incorporated mutations and deletions that were then introduced into viable genomes by recombination. Such a very unlikely course of events and its biological consequences would be difficult to distinguish from variations occurring directly in viable genomes.

The mutant spectrum of the progeny of the third-generation biological sub-clones exhibited a pattern of dominance of mutations in the 0.10%–0.49% frequency range that mirrors the distribution previously identified with nasopharyngeal isolates of SARS-CoV-2 (19, 20). The dominance of low-frequency mutations may be yet another indication of the adverse effect on viral fitness that mutations have for a large genome virus (2, 3, 71). Point mutations that were detected at low frequency in the mutant spectrum of USA-WA1/2020 were present in the consensus sequence of some biological sub-clones (compare Fig. 2 and Table S2 in https://saco.csic.es/s/kYsz6A4sbzssZRp), denoting easy mutation frequency transits during the evolution of the virus. Among the low-frequency amino acid substitutions in nsp12, L514P (in subclone c11.2.1g) and L727P (in subclone c10.2.2g and USA-WA1/2020) were previously described in viruses from patients of the Madrid cohort and may have functional effects (18).

The most abundant mutation type in the consensus sequences of the biological clones and sub-clones was C → U in agreement with most other studies (20, 73–75). In contrast, the most abundant mutation types in the mutant spectra of the biological clones and sub-clones were U → C and A → G (*P* < 0.001; proportion test), coincident with those in virus from nasopharyngeal isolates (19). The mutation frequency difference between mutant spectra and consensus sequences could not be due to some artifact derived from reaching the 0.1% mutation frequency cut-off in the ultra-deep sequencing procedure, since the same preferences were observed when the cut-off was set at 0.5% frequency (18). As analyzed in the present study, a consensus sequence is a weighted average of the individual genomic sequences that compose the sample of the population. The mutant spectrum is quantified with a limited number of individual molecules represented in specific amplicons from the sample. Therefore, a possibility is that, on average, U → C transitions inflict a more severe fitness cost than C → U transitions on the genomes harboring them. Such fitness cost would result in an average decrease in the frequency of genomic regions that include U → C transitions, therefore losing weight in their contribution to defining the consensus sequence of the entire quasispecies. This possibility is supported by the known adverse effect on protein and RNA structure of A → G and U → C transitions vs other mutation types (76, 77). In agreement with this proposal, the mechanism of an FMDV polymerase substitution that conferred upon the virus resistance to the mutagenic base analog 5-fluorouracil was to diminish the proportion of A → G and U → C mutations during viral genome replication, the type of mutations most frequently evoked by the analog (78). A non-exclusive possible factor that may contribute to the abundance of G → A and C → U transitions in the consensus sequences is that part of these mutation types may be contributed by cellular editing activities; the editing enzymes may act preferentially on the most abundant viral RNA molecules, which are those with more weight to define the consensus sequence. Despite A → G transitions being compatible with the involvement of adenosine deaminase acting on RNA (ADAR) editing (79–81), in most sub-clones, G was the most frequent residue at the 5′ side of a mutated A (Table S7 in https://saco.csic.es/s/kYsz6A4sbzssZRp), which is a non-preferred 5′ neighbor of the edited positions. Therefore, although it cannot be excluded that some of the A → G transitions might be due to A to I ADAR editing, it is unlikely that the latter was the major driver of SARS-CoV-2 diversification. This is further reinforced by the low template-copying fidelity of the viral polymerase (82), which is expected to contribute point mutations even assuming that the Exo N was as active as in the coronavirus murine hepatitis virus (30). Given the many unknowns related to factors that may influence replicative accuracy and fitness, the origin of the difference in mutation type preferences reflected in SARS-CoV-2 consensus genomic sequences vs mutant spectra of specific amplicons remains an open question.

The mutant spectrum of biological sub-clones includes clade-discordant residues (those that were used to define the consensus sequence of other SARS-CoV-2 clades but not of the USA-WA1/2020 clade), previously observed also in clinical isolates (21, 64). The probability that the presence of clade-discordant residues is the result of chance is negligible (21). In addition to implementing strict procedures to prevent cross-contaminations among viral and RNA samples (described in references 20, 21 and detailed in Materials and Methods), the present study adds new proof of the authenticity of such clade-discordant residues. Indeed, the latter in the biological sub-clones belonged to Alpha, Beta, Gamma, Lambda, and Omicron lineages that were not in circulation when the USA-WA1/2020 sample was obtained or when it was received in our laboratory (https://www.who.int/activities/tracking-SARS-CoV-2-variants). This excludes that clade discordances in USA-WA1/2020 are due to unnoticed co-infections in the patients from whom the virus was sampled (21). The results favor the view that clade discordances are a consequence of the limited occupation of sequence space by SARS-CoV-2 in the course of its evolution. This opens the interesting possibility that the application of predictive algorithms (83, 84) (among other approaches) using mutant spectrum sequences as data input may anticipate likely sequence stretches in future isolates. The widespread occurrence of clade-discordant residues in mutant spectra represents a challenge to ascertain that their presence in clinical isolates is the result of co-infection of patients in periods of co-circulation of different SARS-CoV-2 lineages (85). Two precedents of sequence space occupation restrictions may be mentioned: (i) the long-term antigenic variation of FMDV involved residue alternations (rather than linear accumulation of different substitutions) at key antigenic sites (86). (ii) When low-fitness FMDV biological clones were allowed to regain fitness, their mutant spectra contained mutations that acquired dominance at later stages of evolution of the virus (87); the term “harbinger” was suggested to refer to mutations with predictive value (71). Some amino acid substitutions, however, such as nsp2:R463S, nsp6:T6I, nsp12:S363N, S:D215G, S:H245R, and S:R682W, which were present in the consensus sequence of several clones and sub-clones, are underrepresented in data banks (less than 0.5% worldwide cumulative prevalence in https://outbreak.info/ [88]).

Two objections to our approach to measuring variations in viable SARS-CoV-2 genomes may be presented: (i) the inadequate choice of Vero E6 cells because they tend to mutate the furin cleavage site in protein S (amino acids 681–685) (89–91), and (ii) the limited value of quantifying mutations and deletions in viable genomes because even if modifications were introduced in defective genomes, they could be transferred into viable genomes by recombination (65–69).

Regarding (i), in the consensus sequences determined for biological clones and sub-clones, only one out of four amino acid substitutions (R682W), and one of two deletions (residues 23594–23629, which correspond to amino acids 678–689) mapped within the furin cleavage site (Fig. 2). Moreover, none of the 58 S substitutions in the mutant spectrum of the sub-clones mapped within the furin cleavage site (Table S2 in https://saco.csic.es/s/kYsz6A4sbzssZRp). We cannot exclude that substitutions away from the furin cleavage site may have also been selected in Vero E6 cells (89). The higher ratio of non-synonymous to synonymous mutations in the S-coding region as compared with the nsp12-coding region (Fig. S5; Table S8 in https://saco.csic.es/s/kYsz6A4sbzssZRp) may be also explained by the tolerance of surface proteins to accept amino acid substitutions, which lead to antigenic variation in the absence of immune selection (92, 93). Any cell type imposes constraints on viruses (“to culture is to disturb” [94]), including examples of cell culture-mediated rapid selection of multiple viral mutations (95, 96). No difference in the fitness increase associated with S substitution N501Y was noted when measured using Vero E6, Calu-3, or HAE cells (97). There is no evidence to support that the estimates of viable variant progeny would differ in other cell lines (compare the total number of different mutations scored in the S vs other genomic regions in Fig. 2 and 3 and Table S2 in https://saco.csic.es/s/kYsz6A4sbzssZRp). In fact, given the prior history of passages in Vero cells of the USA-WA1/2020 (53) (see Materials and Methods), sequence modifications would have been more likely selected if the virus had been shifted to a different cell line to perform our study (71). Regarding (ii), the occurrence of mutations in viable genomes that maintain their viability without substantial effect on viral fitness has an immediate potential impact on adaptability. Their transfer from DVGs to infectious genomes would depend on chance recombination events.

In summary, the viable complement of SARS-CoV-2 clonal populations is less heterogeneous than other RNA viruses for which comparable measurements are available. Biological clones rapidly evolve into complex mutant spectra that include clade-discordant residues. The results converge into suggesting considerable limitations in SARS-CoV-2 sequence space—limitations that, however, do not seem to preclude remarkable adaptability of this emergent pathogen (2–5).

## MATERIALS AND METHODS

### Virus, infections, and preparation of biological clones

SARS-CoV-2 isolate USA-WA1/2020 is from the first patient diagnosed with COVID-19 in the USA (53). Following collection of nasopharyngeal and oropharyngeal samples, limiting dilutions of the virus were applied to suspended Vero CCL-81 cells and incubated at 37°C and 5% CO_2_ until cytopathology was observed. The RNA was extracted and sequenced. The virus was passaged two additional times in Vero CCL-81 cells. A final passage in Vero E6 cells provided the preparation that was received in our laboratory from BEI Resources (NIAID, NIH; catalog No. NR-52281; contributor: Centers for Disease Control and Prevention, Atlanta, Georgia, USA; www.beiresources.org); this virus had a consensus genomic sequence identical to that of the initial clinical sample (GenBank accession number MN985325). We prepared a working stock by infecting 3 × 10^6^ Vero E6 with 3 × 10^3^ PFUs of the USA-WA1/2020 from BEI Resources in the BSL-3 facility at Centro de Biología Molecular Severo Ochoa (CBM); the titer of the working stock was 1 × 10^7^ PFU/mL. Aliquots were kept at −80°C in the BSL-3 facility until use.

To obtain biological clones c10, c11, c12, and c13, 1,000 PFUs of USA-WA1/2020 were incubated with 0.01% sodium deoxycholate (Merck) in Dulbecco’s modified Eagle medium (DMEM) for 10 min at room temperature (Table S1 in https://saco.csic.es/s/kYsz6A4sbzssZRp). Then, the mixture was diluted 10-fold in DMEM and plated on Vero E6 cell monolayers (1 × 10^6^ cells in a well M6 plate) in DMEM, 1% fetal bovine serum, 1% DEAE dextran, 25 mM HEPES, and 0.5% agar (Sigma); plaques were allowed to develop for 72 h. The average size plaque was 1 mm in diameter; since the diameter of an M6 plate is 3.5 cm, a viral plaque resulted from the infection of about 800 cells. Material from well-isolated plaques of average size was punched and resuspended in 500 µL of DMEM; 140 µL was used for RNA extraction, quantification, and determination of the consensus genomic sequences using COVIDSeq; 50 µL was used for titration of infectivity, and 90 µL was treated with 0.01% sodium deoxycholate and plated to obtain the second generation sub-clones; identical procedures were followed to obtain and characterize the third-generation sub-clones. Infectivity of USA-WA1/2020 and of its derived biological clones and sub-clones was determined by plating serial dilutions of the viral preparations on Vero E6 monolayers and expressed in PFU per milliliter.

The kinetics of infectious progeny production were determined by infecting 3 × 10^6^ Vero E6 cells with 3 × 10^3^ PFUs of either USA-WA1/2020 (in triplicate) or the progeny of each of the 10 third-generation sub-clones. Infectivity was titrated at 0 h, 2 h, 4 h, 8 h, 24 h, 48 h, and 72 h post-infection. Vero E6 cell monolayers were mock-infected, as negative control in infections and titrations of infectivity.

### RNA extraction, SARS-CoV-2 RNA amplification, and DNA quantification

Total RNA was extracted from 140 µL of cell culture medium using QIAmp Viral RNA Mini Kit 250 (QIAGEN), following the instructions of the manufacturer. Each extraction was carried out separately. Negative controls were run in parallel with each extraction to ensure the absence of contamination with undesired templates. For each amplification, 5 µL of the RNA extract was mixed with 2 µL of each of the forward and reverse primer solutions (to yield 2 ng/µL), 10 µL of 5× buffer, and 1 µL polymerase, using the Transcriptor One-Step RT-PCR kit (Roche Applied Science). Amplifications in the absence of RNA were run in parallel as negative control. To ascertain that the amount of template RNA was not limiting, amplification reactions were carried out with dilutions 1:10, 1:100, and 1:1,000 of each RNA preparation; we proceeded with the sequencing protocol using the undiluted RNA sample only when dilution 1:1,000 yielded a visible DNA band in the gel electrophoresis analysis (2% agarose, with Gene Ruler 1 Kb Plus DNA Ladder from Thermo Fisher Scientific as molar mass standard). The amplified, gel-fractionated DNA was purified employing the QIAquick Gel Extraction Kit (QIAGEN), quantified with the Qubit dsDNA Assay Kit (Thermo Fisher Scientific), and quality-tested using the TapeStation System.

### Quantification of viral RNA

Quantitative real-time PCR (RT-qPCR) of SARS-CoV-2 RNA was carried out using a Light Cycler RNA Master SYBR green I kit (Roche). The nsp12 (polymerase)-coding region of the SARS-CoV-2 genome was amplified using primer oligonucleotides nsp12-CoV2-u14890 and nsp12-CoV2-d15320 (Table S9 in https://saco.csic.es/s/kYsz6A4sbzssZRp). Quantification was relative to a standard curve obtained with known amounts of SARS-CoV-2 RNA synthesized by *in vitro* transcription from plasmid pGEM-T-nsp12-14511-15717 (with inserted cDNA corresponding to residues 14511–15717 of the SARS-CoV-2 genome; numbering according to Wuhan-Hu-1 NCBI reference sequence NC_045512.2). The specificity of the reaction was tested with the denaturation curve of the amplified DNA. Negative controls (without template RNA) were run in parallel with each amplification reaction to ascertain the absence of contamination with undesired templates.

### Precautions and controls to prevent cross-contaminations among samples

Concerning procedures and controls to prevent cross-contamination during laboratory handling of the viral and RNA samples, the following measures were established. Care was taken to avoid cross-contacts in the course of sample collection at the Fundación Jiménez Díaz Hospital and during transport to the BSL-3 laboratory at CBM. In the laboratory, each sample was dealt with separately (only one sample in the cabin at any given time) in high containment Class II, type A biological safety cabins that conform with European regulation EN-12469; cabins were subjected to UV irradiation after use. In a control experiment, an open tube containing water was kept in the cabin during successive extractions of nasopharyngeal swabs. Then, the contents of the open tube were subjected to the standard RT-PCR amplification protocol; no DNA product was obtained. Non-disposable laboratory materials (including micro-pipette bodies) were disinfected employing a VIRKON (Rely+On Virkon LanXess) solution. Reagents for RT-PCR amplifications were prepared in an isolation cabin located in the standard biochemistry laboratory (where no SARS-CoV-2 samples are stored). Negative RT-PCR amplification controls consisting of a sample with all the reagents except RNA were included for each amplicon and amplification reaction. The experiments were carried out using low-binding barrier sterile tips (Corning). No evidence of contamination was obtained in any of the control assays. Following the established protocol, during library preparation of DNA for ultra-deep sequence analysis, amplicons were indexed to identify the set of sequences for each sample.

For all these considerations regarding precautions taken for sample collection and transport, biosafety measures, and experimental controls, laboratory contamination accounting for the presence of shared genomic changes among samples or of clade-discordant residues in SARS-CoV-2 mutant spectra is exceedingly unlikely (additional arguments are given in references 20, 21).

### Determination of consensus sequences

Consensus genomic sequences of the preparation of USA-WA1/2020 and of the biological clones and sub-clones were obtained using COVIDSeq with the MiSeq platform of Illumina. Quality check (QC) was performed with BaseSpace (Illumina). As a filter for quality control, only sequences scored as “good” in the QC score were accepted. The genomic sequence of USA-WA1/2020 was aligned with reference sequence Wuhan-Hu-1 (NC_045512.2), using the Bio-IT Processor (version: 0 × 04261818). The mutations that distinguish the two sequences are C8782T (synonymous) in nsp4, C18060T (synonymous) in nsp14, and T28144C (L84S) in ORF8. Point mutations and deletions in the consensus sequence of viral clones and sub-clones were counted relative to the sequence of USA-WA1/2020.

### Ultra-deep sequencing

Four amplicons of the nsp12 (polymerase)-coding region (spanning genomic residues 14511–16075) and six amplicons of the spike (S)-coding region (spanning genomic residues 21424–23666) (numbering according to Wuhan-Hu-1 NCBI reference sequence NC_045512.2) were obtained from the RNA extracted from 10 biological sub-clones, using the primers listed in Table S9 and Fig. S6 (https://saco.csic.es/s/kYsz6A4sbzssZRp). Each preparation was adjusted to a DNA concentration of 1.5 ng/µL, indexed using the SeqCap Adapter kit A/B (Roche), and processed with the Kapa Hyper Prep kit (Kapa Biosystems, Roche). DNA was quantified with the LightCycler 480 system. The deep sequencing was performed using the MiSeq Illumina platform and the MiSeq Reagent kit v3 (2 × 300 bp mode; 600 cycle kit). The sequences were presented as Fastq data and were processed with the SeekDeep bioinformatics pipeline (98), using options: --extraExtractorCmds=-- checkRevComplementForPrimers –primerNumOfMismatches 3” “—extraProcessClusterCmds=--fracCutOff 0.001 –rescueExcludedOneOffLowFreqHaplotypes.” Mutations and deletions were detected with a frequency cut-off of 0.1%.

### Data and controls to ascertain ultra-deep sequencing trustworthiness

Reliability of the point mutations, deletions, and haplotypes identified with a frequency cut-off of 0.1% in the sub-clonal populations is based on the following sequencing outputs, their interpretation, and other controls: (i) the average clean read coverage was 117,963 (range 36,172–242,148; Table S10 in https://saco.csic.es/s/kYsz6A4sbzssZRp). (ii) A total of 92.2% of the residues that were sequenced yielded a quality score *Q* > 30, as calculated by the Illumina platform (https://emea.illumina.com/systems/sequencing-platforms/miseq/specifications.html). In addition, the experimental procedures and the bioinformatics pipelines used in the present investigation are identical to those of our previous studies (19, 20), where we gathered the following additional observations. (iii) Out of 97 mutations, 96 mutations and the 10 deletions that were identified with a 0.5% mutation frequency cut-off in the mutant spectrum of patients’ SARS-CoV-2 were also detected with the 0.1% cut-off. (iv) Mutation types and their distribution among the first, second, and third codon positions were the same with the mutation frequency cut-off either at 0.5% or at 0.1%. (v) The percentage of amino acid substitutions found with the 0.5% and 0.1% frequency cut-off that were represented in the GISAID data bank was very similar. (vi) Equally similar were the acceptability and predicted functional effects of amino acid substitutions scored with 0.5% and 0.1%, as judged from the PAM250 substitution matrix and the SNAP2 predictor, respectively; it is unlikely that the similarity of functional effects would be maintained if the majority of substitutions obtained with the 0.1% cut-off were artifacts. (vii) The number of mutations scored upon lowering the point mutation frequency cut-off from 0.5% to 0.1% increased 55-fold for the nsp12 (polymerase)-coding region and 97-fold for the S-coding region; it is unlikely that the increase in the number of artifactual mutations was different in the two genomic regions examined with identical experimental and bioinformatics protocols. (viii) Duplicate sequencing of six amplicons of the virus from eight COVID-19 patients yielded a similar number of point mutations and their frequency (*r* = 1, *P* < 0.0001 using the Pearson correlation test); in addition, duplicate amplifications and sequencing of three nsp12 amplicons of the reference isolate USA-WA1/2020 also yielded a similar result (*r* = 1, *P* < 0.0001 using the Pearson correlation test). (ix) The most frequent mutation types in our SARS-CoV-2 analyses are U → C and A → G, while the errors in the Illumina system are mainly A ↔ C and G ↔ T (99). None of the observations (i) to (ix) would hold if a substantial proportion of mutations recorded with the 0.1% frequency cut-off were artifacts (for further detail see references 5, 19, 20).

### Statistics

The statistical significance of differences in infectivity, RNA concentration, and specific infectivity was calculated using the one-way ANOVA, while differences in growth kinetics were determined using the two-way ANOVA. In both cases, Tukey’s *post hoc* multiple pairwise comparison test was applied using GraphPad Prism 8.00. The statistical significance of differences between the number of mutations was determined using the proportion test, using R software version 4.0.2. Differences between diversity indices were determined with the Kruskal-Wallis test, followed by Dunn’s multiple comparison test using GraphPad Prism 8.00.

## Data Availability

The data presented in the study have been deposited in NCBI/ENA/DDBJ under accession number PRJEB85704.
